# Molecular epidemiology of chicken anemia virus in commercial farms in China

**DOI:** 10.1186/1743-422X-8-145

**Published:** 2011-03-30

**Authors:** Yassir M Eltahir, Kun Qian, Wenjie Jin, Pingping Wang, Aijian Qin

**Affiliations:** 1 Ministry of Education Key Lab for Avian Preventive Medicine, Yangzhou University, Yangzhou, 225009, PR China; 2 Department of Preventive Medicine and Veterinary Public Health, Faculty of Veterinary Science, University of Nyala, Nyala, Sudan

## Abstract

**Background:**

Chicken anemia virus (CAV) is the causative agent of chicken infectious anemia (CIA). A high prevalence of CAV has been reported in China. However, VP1 sequences of Chinese isolates show no clear genotype clustering or correlation with geographic origin. Therefore, the present study aimed to detect and characterize CAV isolates from China based on sequence and phylogenetic analysis of the VP1, VP2 and VP3 genes.

**Results:**

Of 460 spleen samples tested by PCR, 47 (10.22%) were found to be positive for CAV. A total of 25 CAV, approximately full genomes, from different commercial farms were characterized. Phylogenetic analysis of the Chinese CAV sequences together with strains from different countries resulted in four distinct groups (A-D) with significant high bootstrap values. The Chinese viral sequences were located as four different clusters within groups A and D. All the Chinese CAV genomes characterized in this study had glutamine (Q) at amino acid position 394, which indicated that all are highly pathogenic. Mutations associated with attenuation and weaker reactivity with monoclonal antibody 2A9 were absent in the Chinese sequences.

**Conclusions:**

We revealed that CAV prevalence was lower than that reported previously in commercial farms in China. We also showed four distinct sequence groups (A-D), and genetic variability in local CAV sequences that could be divided into four groups based on phylogenetic analysis.

## Background

Chicken anemia virus (CAV), a member of the family Circoviridae, is a non-enveloped, icosahedral virus with a negative-sense, single-stranded circular DNA genome [1]. The CAV genome consists of 2.3 kb, with three partially overlapping open reading frames (ORFs) for VP1, the major viral structural protein (51.6 kDa); VP2, a scaffolding protein (24 kDa); and VP3, a non-structural protein named apoptin (13.6 kDa) for its ability to induce apoptosis; VP1 and VP2 are the main targets of neutralizing antibodies[2]. The VP1 gene has the highest variability of the three overlapping ORFs, according to sequences that have been submitted to GenBank [3].

To date, all viruses seem to belong to the same worldwide serotypes. However, because there are currently only a few full genome sequences available for CAV strains from the USA, Asia, Australia and Europe, the emergence of new serotypes cannot be excluded, which would have important consequences for vaccine efficacy and serodiagnosis [4].

In China, CAV was first isolated in 1996 from 25-40-day-old broilers [5]. A survey in domestic poultry in farms in 5 Chinese provinces (Beijing, Guangdong, Zhejiang, Shanghai, and Tianjin Shi) showed a 42% overall seroprevalence [6]. On the other hand, in Southeast China, studies undertaken on live bird markets also indicated a high prevalence (87%) of the virus [7].

Although considerable numbers of VP1 sequences from China are available in GenBank, to the best of our knowledge, no systematic full genome analysis of Chinese strains has been performed. Here, we report the detection and characterization of CAV genomes based on sequence and phylogenetic analysis of the entire coding regions (VP1, VP2 and VP3) of the genome from commercial broiler and layer breeder chickens in China.

## Methods

### Samples

Between April and November 2010, a total of 350 spleen samples were collected from diseased chickens, aged 6-36 weeks, during necropsy at veterinary hospitals in Anhui (*n *= 51),Fujian(*n *= 14), Hunan (*n *= 127) and Jiangsu (*n *= 158) provinces. In parallel, 110 spleen samples were collected from 1-7-day-old chickens from four different commercial farms from Jiangsu province. Chickens originated from 22 different flocks on commercial farms. Flocks comprised 900-30 000 chickens, and none of the farms were vaccinated against CAV.

### DNA extraction

According to the manufacturer's instructions, DNA was extracted from spleen samples using the commercially available Flexi Gene DNA Kit (Qiagen GmbH, Hilden, Germany). The DNA was then quantitated and stored at -20°C until PCR was performed.

### Virus detection by PCR

The extracted DNA was first screened by PCR for CAV DNA using specific primers, CAV1: 5'-GCA GTA GGT ATA CGC AAG GC-3' and CAV2: 5'-CTG AAC ACC GTT GAT GGT C-3', covering a 186-bp region on the highly conserved VP2 coding gene [2]. The PCR amplification was carried out in PCR buffer that contained 1.5 mM MgCl_2_, 200 μM of each dNTP, 10 *p*mol each primer, and 1.0 U Taq DNA polymerase (Fermentas, Shenzhen, China) in a 25-μl total reaction volume in an automated thermal cycler (Gene Amp PCR System 9700, Applied Biosystems, Foster City, CA, USA) using the following cycling profile: initial denaturation of 94°C for 2 min, followed by 35 cycles of denaturation, annealing and extension at 94°C for 30 s, 60°C for 30 s and 72°C for 1 min, respectively, and the final extension was carried out at 72°C for 7 min. The PCR products were then analyzed by 1.5% agarose gel electrophoresis and imaged with the EpiChem system (UVP Bioimaging Systems, Garland, CA, USA).

### Amplification of the CAV genome

Primers VP1F: 5'-AGCCGACCCCGAACCGCAAGAA-3' and VP1R: 5'-TCA GGG CTG CGT CCC CCA GTA CA-3' were used to amplify the VP1 region, and VP2F: 5'-GCG CAC ATA CCG GTC GGC AGT-3' and VP2R: 5'-GGG GTT CGG CAG CCT CAC ACT AT-3' were used to amplify the VP2 region from PCR-positive samples. These two primer sets covered the entire coding regions of CAV [8]. The PCR amplification was carried out in PCR buffer that contained 1.5 mM MgCl_2_, 200 μM of each dNTP, 10 pmol each primer, and 1.0 U Takara LA Taq™ polymerase (TaKaRa Biotechnology Co., Ltd., Dalian, China) in a 25-μl total reaction volume. The reaction was carried out in an automated thermal cycler (Gene Amp PCR System 9700, Applied Biosystems, Foster City, CA, USA). Amplification of 1390 bp of the VP1 region was carried out with initial denaturation of 94°C for 4 min, followed by 34 cycles of denaturation, annealing and extension at 94°C for 1 min, 60°C for 1 min and 72°C for 2 min, respectively, and the final extension was carried out at 72°C for 15 min. Amplification of 713 bp of the VP2 region was carried out with initial denaturation of 94°C for 4 min, followed by 34 cycles of denaturation, annealing, extension at 94°C for 1 min, 63°C for 1 min and 72°C for 1 min, respectively, and final extension was carried out at 72°C for 5 min. The PCR products were then analyzed by 1.5% agarose gel electrophoresis and imaged with the EpiChem system (UVP Bioimaging Systems). In all PCR reactions, a previously characterized CAV isolate maintained in our laboratory was uses as a positive control. The PCR mixture was used as a negative control. The VP1 and VP2 regions were purified using an agarose gel with the QIAquick Gel Extraction Kit (Qiagen), and sequenced with ABI Prism, BigDye Terminator (Shanghai, China) using PCR primers. For each samples, DNA extraction and PCR was run at least twice.

### DNA cloning and sequencing

In the case of sequencing failure, specific PCR products of the VP1 and VP2 regions were subcloned into pGEM^®^-T Easy vector (Promega, Madison, USA). The ligated products were transformed into Escherichia coli subcloning efficiency DH5α competent cells (Invitrogen, Carlsbad, CA, USA). Colonies that contained DNA inserts of the correct size were picked and grown overnight in 3 ml Luria-Bertani (LB) liquid medium containing ampicillin. The mini-preparation of plasmid DNAs was performed using the plasmid extraction Kit (AxyPrep™ Plasmid Miniprep, Hangzhou, China), following the manufacturer's instructions. The plasmid DNAs were employed as a template for sequencing with ABI Prism, BigDye Terminator.

### Analysis of sequence data

Sequences were analyzed using the Bioedit program [9]. Forward and reverse sequences were aligned with Clustal W [10]. Phylogenetic and molecular evolutionary analyses were based on the VP1, VP2 and VP3 genes sequences, using MEGA version 3.1 [11]. Phylogenic analysis of nucleic acid and deduced amino acid sequences was done with the neighbor-joining method, Kimura 2-parameter model. The amino acid sequences were also analyzed with the neighbor-joining method, with the Poisson correction. Bootstrap values (1000 replications) were indicated on each tree. Nucleotides were numbered according to Meehan et al. [8]. Relevant VP1, VP2 and VP3 sequences available on GenBank were used for comparison.

## Results

### Virus detection

**Figure 1:**
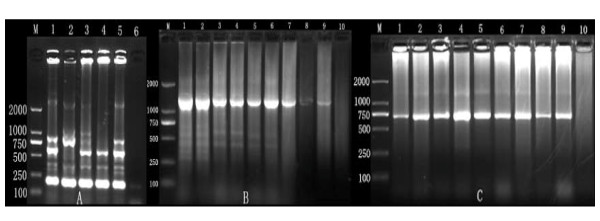
**Detection of CAV by PCR and amplification of CAV  VP1 and VP2.** (A) Specific PCR product (186 bp) detected in CAV-infected birds; (M) 2 kbp DNA ladder marker (TaKaRa). Lane 1, positive control; lanes 2-5, positive samples; lane 6 negative control; (B) PCR amplification of CAV VP1 by PCR; (M) 2 kbp DNA ladder marker (TaKaRa). Lane 1, positive control; lanes 2-9, amplified VP1 (1390 bp); lane 10, negative control; (C) PCR amplification of CAV VP2 by PCR; (M) 2 kbp DNA ladder marker (TaKaRa). Lane 1, positive control; lanes 2-9, amplified VP 2 (731 bp); lane 10, negative control.

Of the 460 spleen samples studied, specific PCR products (186 bp) of the VP2 region were detected in 47 (10.22%) adult birds (6-36 weeks old), with 6, 17 and 24 from Anhui, Hunan and Jiangsu provinces, respectively (Figure 1A). In comparison, none of the samples collected from 1-7-day-old chickens was positive by PCR.

### PCR amplification of different regions of CAV genome

PCR amplification of the VP1 region using primers VP1F and VP1R yielded a specific product of 1390 bp, and similarly, PCR amplification of the VP2 region using primers VP2F and VP2R yielded specific product of 713 bp (Figure 1B and 1C). The authenticity of PCR amplification was confirmed by the expected size in agarose gel cloning and sequencing. Twenty five CAV genome sequences characterized in this study were submitted to GenBank with accession numbers [HQ872023-HQ872047]. Identical sequences were submitted only once.

### Sequence alignment and phylogenic analysis

Nucleotides of the 25 Chinese viral genomes were annotated, and a length of 1766 bp that contained almost the entire CAV coding region except for a few amino acids at the C terminus of VP1 was obtained.

The maximum Kimura distance between the new 25 Chinese VP1, VP2 and VP3 sequences was 4.92% between JS-China 60 and JS-China 14, in comparison with 5.17% between Australia EF683159 and USA L14767 for the world maximum Kimura distance calculated from worldwide relevant VP1, VP2 and VP3 sequences (neighbor-joining method, Kimura 2-parameters model).

The Chinese viral sequences presented 8 nucleotide mutations in the VP3 protein, which had not been observed previously in strains from other parts of the world (nt positions 37, 57, 66, 131, 134, 135, 141 and 145), of which, seven were found in more than one sequence. There were 10 mutations (nt positions 9, 15, 19, 21, 54, 106, 107, 273, 316 and 474) in the VP 2 protein, of which, three were found in more than one sequence. Finally, there were 60 mutations in the highly variable VP1 protein, of which, 36 were found in more than one sequence (nt positions 12, 21, 26, 30, 49, 73, 210, 219, 225, 231, 252, 288, 324, 423, 567, 600, 649, 661, 669, 681, 774, 786, 797, 867, 876, 882, 913, 924, 939, 943, 945, 1011, 1012, 1023, 1033 and 1044); numbered according to a previous study [12].

**Figure 2:**
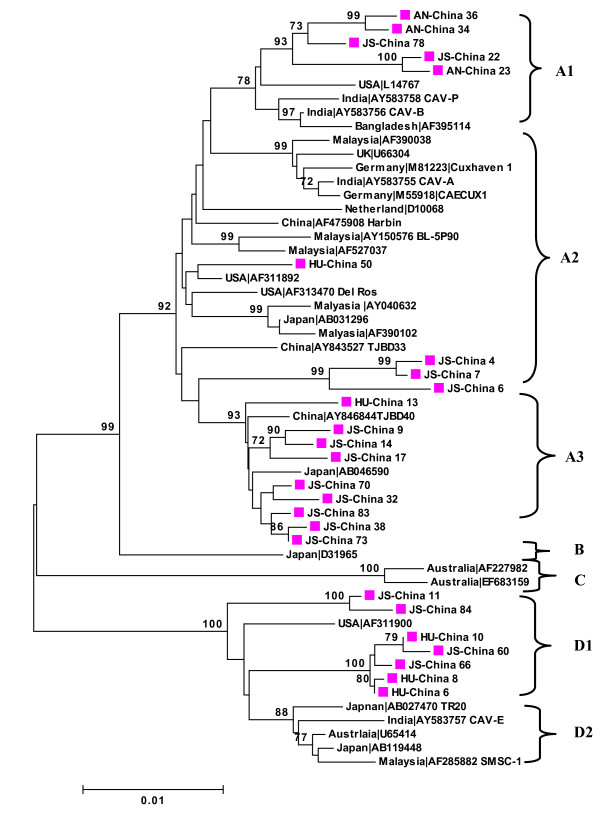
**Phylogenetic analysis of the nucleic acid sequence of 25 new complete VP1, VP2 and VP3 sequences from Anhui (AN), Hunan (HU) and Jiangsu (JS) provinces, China and 29 relevant VP1, VP2 and VP3 sequences currently available in GenBank**. Values ≥70 are indicated on the branches (as percentages). Sequences from the present study (colored closed symbols) are named as follows PP-China where PP is the area of origin. Sequences from GenBank were given the country name followed by accession number. The four major groups were identified as A, B, C and D.

Phylogenetic analysis at the nucleotide level (1766 bp) of the Chinese CAV genomes characterized in this study, together with CAV strains from different countries, led to four distinct sequence groups (A, B C and D), with significant high bootstrap values of 92, 99 and 100 separating them, respectively. Group A could be further divided into subgroups A1, A2 and A 3 that were separated from each other by a bootstrap value of 78 and 93. Group D could be further divided into subgroups D1 and D2 that were separated from each other by bootstrap value of 88. The new Chinese CAV sequences were located as four different clusters within groups A and D (Figure 2).

Group A represented a major group of 39 isolates that originated from Asia, the USA and Europe, in which, five new CAV sequences from China (JS-China 22, JS-China 78, AN- 23, AN-China 36 and AN-China 34) were located within subgroup A1, together with other isolates from the USA, India and Bangladesh.

Four new Chinese sequences (HU-China 50, JS-China 4, JS-China 7 and JS-China 6) were clustered within subgroup A2 that contained the prototypic CAV strain CAE CUX1 from Germany, together with other isolates that originated from India, Malaysia, Japan, Bangladesh, UK, Netherlands and USA (Figure 2).

The majority of Chinese CAV genomes characterized in this study (HU-China 13, JS-China 9, JS-China 14, JS-China 17, JS-China 70, JS-China 32, JS-China 83, JS-China 38 and JS-China 73) and three previous characterized Chinese isolates (AY846844 TBJ 40, AY 843527 and AF 475908) were clustered within subgroup A3 together with one isolate from Japan (Figure 2).

Only one isolate from Japan (Japan AB 046590) and two from Australia (Australia AF227982 and Australia EF683159) comprised group B and C, respectively.

Seven new Chinese sequences (JS-China 11, JS-China 84, JS-China 60, JS-China 66, HU-China 6, HU-China 8 and HU-China 10), together with one isolate from USA comprised subgroup D1. Other isolates from India, Malaysia, Japan and Australia comprised subgroup D2 (Figure 2).

### Amino acid alignment and phylogenetic analysis

When the amino acids of the 25 Chinese sequences were aligned, the maximum Kimura distance was increased to 2.92% between JS-China 60 and HU-China 13, in comparison with 2.5% between JapanD31965 and MalaysiaAF285882 SMS1 and USA 4903007 TR 20.

**Figure 3:**
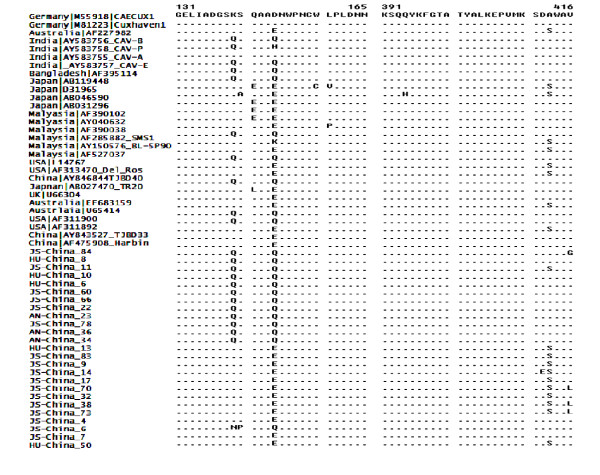
**Amino acid alignment of highly variable region of VP1 coding sequences of different CAVs (aa 131-165) and the major genetic determinant of CAV virulence (aa 391-416)**. Sequences are compared with the Cux 1 isolate. Consensus with Cux 1 is indicated as dots and differences are indicated by the single-letter code.

Analysis of the deduced 189 amino acids of VP3 protein revealed only two amino acid variations detected in a single genome, which were specific to Chinese sequences (aa 32 and 56). The deduced 216-amino-acid sequence of VP2 protein revealed 8 variable amino acids that were specific to the Chinese sequences detected in more than one viral genome (positions 36, 160, 171, 178, 181, 203, 204 and 206), and a additional three substitutions detected in a single genome (aa 106, 152 and 204). In contrast, the deduced 431-amino-acid sequence of VP1 protein revealed 14 variable amino acids that were specific to the Chinese sequences detected in more than one sequence (aa 13, 22, 45, 76, 92, 182, 294, 332, 370, 371, 379, 381, 412, 416 and 429), and an additional 16 substitutions that were detected in single genome (positions 49, 65, 66, 67, 139, 140, 197, 219, 279, 282, 283, 288, 313, 380 and 416). The amino acid at position 394 was glutamine (Q) in all Chinese sequences under the study; numbered according to a previous study [12] (Figure 3).

**Figure 4:**
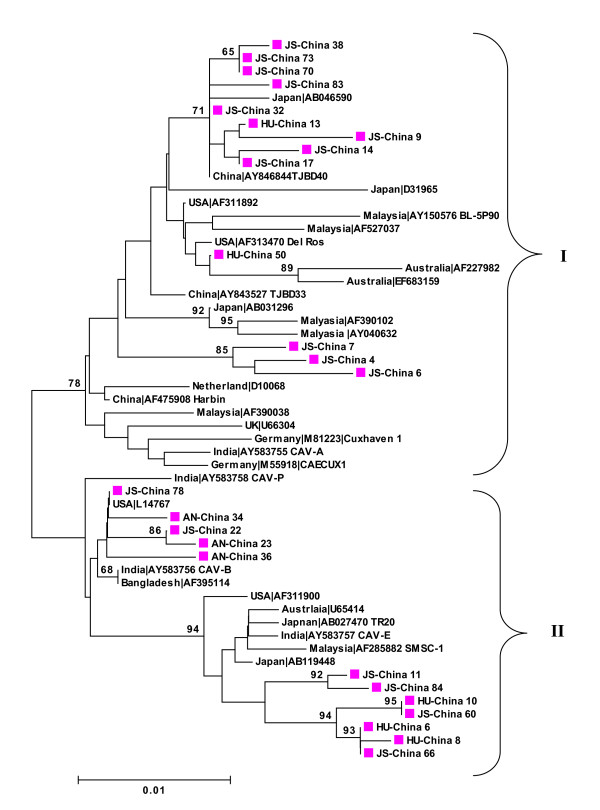
**Phylogenetic analysis of the amino acid sequence of 25 new complete VP1, VP2 and VP3 sequences from Anhui (AN), Hunan (HU) and Jiangsu (JS) provinces, China and 29 relevant VP1, VP2 and VP3 sequences currently available in GenBank**. Values ≥70 are indicated on the branches (as percentages). Sequences from the present study (colored closed symbols) are named as follows PP-China where PP is the area of origin. Sequences from GenBank were given the country name followed by accession number. The two major groups were identified as I and II.

The phylogenetic tree at the amino acid level did not support the grouping and subgrouping of sequences as established by nucleotide sequences. Instead only two major groups each of which contains Chinese isolates could be developed (Figure 4).

Five new Chinese CAV sequences (JS-China 78, AN-China 34, JS China-22, AN-China 23 and AN-China 36), together with isolates from USA (USA L14767), India (AY 583756 CAV-B and AY 583758 CAV-P) and Bangladesh (AF 395114) that clustered in group A at the nucleotide level became closely related to group D at the amino acid level. Similarly, two strains from Australia (Australia AF227982 and Australia EF683159) and one isolate from Japan ( Japan D 31965) that belonged to group C and B, respectively, at the nucleotide level became closely related to group A at the amino acid level (Figure 4).

## Discussion

CAV is an economically important pathogen worldwide due to its highly immunosuppressive effect. In China, CAV was first reported in 1996 [6]. Later, the VP1 protein sequences that were amplified from isolates from live bird market in Southeast China showed no clear genotype clustering or correlation with geographic origin [7]. Therefore, the need was felt for further molecular characterization using long sequences of these viruses to establish the variations among them, if any, which would help to devise a suitable control strategy to prevent losses in the poultry industry.

The present study is believed to be the first molecular analysis of approximately full-length CAV genomes from China. The study was based on the genetic diversity of the VP1, VP2 and VP3 of 25 new CAV genome sequences.

Of the 460 spleen samples screened by PCR, CAV genome was detected in 47 adult birds only (10.22%), but it was not detected at all in young birds (1-7 days old). This low prevalence as compared with the previous 42% seroprevalence [6] and the high nested PCR detection of 87% [7] could have been due to the difference in the type of sample used, areas of study, and the sensitivity of detection methods conducted in each study [13]. After about 3 weeks of age, immunocompetent chickens are resistant to disease, but they can acquire asymptomatic infections and transmit the virus horizontally [3-14]. The adult birds tested in this study were probably subclinically infected. Thus, detection of CAV genome in adult birds alone indicates that horizontal transmission plays a major role in CAV infection in China, and that young birds are partially protected by maternally derived antibodies [7].

At the nucleotide level of the 1766 bp from position 386 to 2151 [12], the new Chinese viral sequences showed 4.92% variation among them, which revealed that they were unique, because the maximum variation among CAV isolates from other countries was 5.1% [15].

Phylogenetic analysis of CAV strains allowed easy grouping of the sequences into four distinct groups (A-D) and five subgroups (A1, A2, A3, D1 and D2) with significantly high bootstrap values. Previous phylogenetic analysis based on the full genome of only 13 CAV isolates from different countries also categorized CAV strains into four groups (A-D), but no subgroups were reported [16]. In contrast, studies using only the VP1 sequences from different parts of the world have demonstrated that CAV isolates could be grouped into three major clusters (I, II, IIIa and IIIb). However, this clustering system was not fulfilled later when 74 VP1 CAV sequences from China were included [7]. In the present study, the amplified 1766 bp from the CAV genome provided a clear genotype clustering of CAV isolates into four major groups. However, no correlation with the geographic origin of the CAV isolates was obtained for groups A and D despite the use of satisfactory sequences data. In contrast, groups B and C comprised only isolates that originated from Japan and Australia. Thus, for better understanding of the molecular epidemiology of CAV isolates, more sequence data are required globally.

The new Chinese CAV sequences were located within subgroups A1, A2 A3 or D1. This indicates that four genetically different CAV clusters are circulating in China. The fact that none of these four clusters was solely established by Chinese isolates reveals that CAV isolates from China might have originated from different parts of the world, namely Europe, Australia, Asia and USA.

The nucleotide mutations observed in the VP3 and VP2 regions of the new Chinese sequences resulted in 6 amino acid variations in the C-terminal quarter of the VP2 protein only. This is in accordance with other studies that have indicated that the N-terminal half of VP3 and the N-terminal three quarters of VP2 are well conserved, and might sustain essential functions of these proteins [8-17].

Analysis of 431 amino acids of the VP1 region indicated 30 amino acid variations that were specific to the Chinese viral genomes. Only two of these variations observed in one sequence were in the highly variable region (aa 139-151). In contrast, no mutations have been reported previously in the VP1 protein of Chinese isolates [7].

The amino acid at position 394 in VP1 was reported to be a major genetic determinant of virulence. CAV isolates are highly pathogenic if its glutamine (Q) or less pathogenic if its histidine (H) [8-18]. All Chinese CAV genomes characterized in this study had glutamine (Q) at this position, which indicated that all were highly pathogenic. Mutations associated with attenuation and weaker reactivity with monoclonal antibody 2A9 (complete I75, T89, L125, L141, E144 or H394) [9] were absent in the Chinese sequences. This observation is in agreement with previous studies on the VP1 of Chinese isolates [7].

The phylogenetic analysis of sequences at the amino acid level did not support the grouping based on the nucleotide sequences. Several studies have shown that groupings of CAV isolates based on nucleotide and amino acid sequences differ as a result of silent mutations, and consequently, CAV isolates are almost identical at the amino acid level despite differences at the nucleotide level [7,16,19].

## Conclusions

This is the first report on the characterization of 25 full genomes of CAV from China. The prevalence of CAV in the area of study was found to be lower than that reported previously in commercial poultry farms from other parts of China. CAV isolates worldwide were clustered into four major groups. Four different, highly pathogenic genotypes of CAV were found to be currently evolving in China. The study provides a basis for future epidemiological research on CAV, although more sequences data are needed globally.

## Competing interests

The authors declare that they have no competing interests.

## Authors' contributions

YME designed the study, carried out the experiment and drafted the manuscript. QA supervised all experiments and participated in the analysis of data with JW, QK and WP, provided discussion and the preparation of the final report. All authors read and approved the final manuscript.
